# Reorganization of Brain Functional Network during Task Switching before and after Mental Fatigue

**DOI:** 10.3390/s22208036

**Published:** 2022-10-21

**Authors:** Hongyang Zhong, Jie Wang, Huayun Li, Jinghong Tian, Jiaqi Fang, Yanting Xu, Weidong Jiao, Gang Li

**Affiliations:** 1 Key Laboratory of Urban Rail Transit Intelligent Operation and Maintenance Technology & Equipment of Zhejiang Provincial, Zhejiang Normal University, Jinhua 321004, China; 2 College of Mathematics and Computer Science, Zhejiang Normal University, Jinhua 321004, China; 3 College of Teacher Education, Zhejiang Normal University, Jinhua 321004, China; 4 Key Laboratory of Intelligent Education Technology and Application, Zhejiang Normal University, Jinhua 321004, China; 5 College of Engineering, Zhejiang Normal University, Jinhua 321004, China; 6 College of Mathematical Medicine, Zhejiang Normal University, Jinhua 321004, China

**Keywords:** mental fatigue, electroencephalogram (EEG), functional connectivity, brain functional network, machine learning, task switching, mental arithmetic task (MAT), 2-back task (2-BT)

## Abstract

Mental fatigue is a widely studied topic on account of its serious negative effects. But how the neural mechanism of task switching before and after mental fatigue remains a question. To this end, this study aims to use brain functional network features to explore the answer to this question. Specifically, task-state EEG signals were recorded from 20 participants. The tasks include a 400-s 2-back-task (2-BT), followed by a 6480-s of mental arithmetic task (MAT), and then a 400-s 2-BT. Network features and functional connections were extracted and analyzed based on the selected task switching states, referred to from Pre_2-BT to Pre_MAT before mental fatigue and from Post_MAT to Post_2-BT after mental fatigue. The results showed that mental fatigue has been successfully induced by long-term MAT based on the significant changes in network characteristics and the high classification accuracy of 98% obtained with Support Vector Machines (SVM) between Pre_2-BT and Post_2-BT. when the task switched from Pre_2-BT to Pre_MAT, delta and beta rhythms exhibited significant changes among all network features and the selected functional connections showed an enhanced trend. As for the task switched from Post_MAT to Post_2-BT, the network features and selected functional connectivity of beta rhythm were opposite to the trend of task switching before mental fatigue. Our findings provide new insights to understand the neural mechanism of the brain in the process of task switching and indicate that the network features and functional connections of beta rhythm can be used as neural markers for task switching before and after mental fatigue.

## 1. Introduction

Task switching refers to a state that converts from one cognitive task to another during mental work, which is an important part of human cognitive control [1]. In general, psychologists believe that when a task is completed, all the materials about it, including perception, attention, response, and memory will be combined into a sampled task set. The changes in task sets for different tasks can lead to competition in the brain’s nerves [2]. Since an individual’s limited brain cognitive resources cannot well accommodate the simultaneous execution of multiple tasks, the brain reallocates cognitive resources when faced with task-switching problems in daily life [3,4,5]. Moreover, it is well known that overuse of brain resources can make the brain mentally fatigued [6,7]. Up to now, the changes in the brain caused by task switching before and after mental fatigue are still unclear.

Over the past few decades, many cognitive science techniques that record physiological signals have been used to measure electrical brain activity. Electroencephalogram (EEG), magnetoencephalogram (MEG), functional magnetic resonance imaging (fMRI), and functional near-infrared spectroscopy (fNIRS) have been widely applied in cognitive neuroscience and psychology due to their ability in detecting brain activity [8,9,10,11]. EEG signals are the sum of the postsynaptic potentials of neurons in the cerebral cortex, and the generated bioelectric signals can be collected by electrode sensors [12]. It has the advantages of low cost, non-invasiveness, high temporal resolution, etc. Multiple studies have proven that EEG is an effective carrier for recording spontaneous bioelectric activity in the brain, and is suitable for assessing brain activity, especially mental fatigue [13,14,15].

Several studies have indicated abnormal changes in the neural mechanism of the brain during task switching [16,17,18]. In recent years, the task-switching paradigms were frequently used to investigate the cognitive control of the brain during task-switching [19,20]. Studies have found that proactive control of the brain is closely related to theta and delta rhythms synchronization activity in the frontal and parietal regions [21,22]. Moreover, Gevins et al. found that lower alpha and theta activities increased in magnitude with task difficulty, when switching from the easy task to the difficult task [16]. Previous studies of task switching have mainly focused on abnormal changes in EEG rhythms and failed to reveal the neural mechanism in task switching from the perspective of brain networks. Brain network features reflect information transfer between different areas of the brain, which can better explain brain activity during task switching [23,24,25]. Besides, the task-switching paradigms frequently used in existing studies cannot well characterize realistic cognitive tasks, and the brain neural mechanism during task switching before and after mental fatigue has not been investigated.

In this work, two mental tasks, 2-BT and MAT were used to perform task switching. MAT is a multi-step computational process that normally involves various cognitive processes. Whereas the 2-BT focuses on continuously updating working memory [26]. Functional connectivity and network features of the delta, theta, alpha, and beta rhythms during two task switching states were extracted and statistical analysis was conducted. The present study constructed an analytical framework to investigate the brain neural mechanism during task switching before and after mental fatigue from the perspective of brain networks. The findings of this work may provide new insights and neural markers for future studies related to task switching. To our knowledge, this study is the first in-depth analysis of task switching before and after mental fatigue using brain functional networks.

## 2. Materials and Methods

### 2.1. Participants

Twenty healthy young volunteers were recruited to take part in this EEG experiment (16 males and 4 females, mean age 28.6 ± 4.62 years old, ranging from 19 to 38 years). All participants were pre-screened to ensure that they were right-handed without any neuropsychiatric disorders, and with normal or corrected-to-normal eyesight. Before the experiment, they were prohibited from taking medications that could affect the results of the experiment and were required to attain sufficient sleep to pledge maximum energy to meet the needs of the experiment. The duration of the experimental task was about 121 min, and the experimental environment was quiet and undisturbed. All participants were given informed consent before the experiment, and the local ethics committee approved the study.

### 2.2. Experimental Design

**Figure 1:**
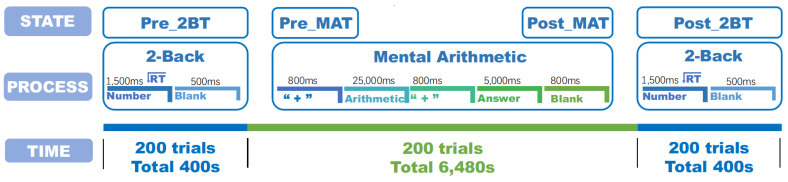
The experimental design. Participants were required to perform three consecutive cognitive tasks for 7280 s. 2-BT was executed once before and after the MAT, respectively. Behavioral data were recorded during 2-BT task execution. The selected data segments were divided into four parts in order of execution time: Pre_2BT, Pre_MAT, Post_MAT, and Post_2BT.

The whole experimental task that each participant needed to perform was composed of three mental tasks. 2-BT was completed first, then MAT, and finally 2-BT. The 2-BT was performed before and after the MAT, respectively (denoted as Pre_2BT, Post_2BT hereafter). There was no time interval between task switching in this experiment. The experimental process is shown in Figure 1.

Participants were presented with a single number on the computer each time during the 2-BT and were required to memorize the number. Each appearance of a number on the screen was a stimulus and the 2-BT required the participants to compare the current stimulus with the last stimulus of the previous one. For each trial, the number was rendered on the screen for 1.5 s, and then the screen was blank for 0.5 s. The Pre_2BT and Post_2BT consisted of 200 trials, lasting 400 s, respectively.

Each MAT trial was to multiply a 1-digit number by a 2-digit number. The 1-digit number was chosen from 6–9, and the 2-digit number was chosen from 60–99. The two numbers multiplied were designed before the experiment, and the difficulty of each trial was similar. For each trial, the participant would be given 0.8 s to memorize the two numbers that need to be multiplied on the screen. The participant would then have 25 s to calculate the result of multiplying the two numbers and 5 s to give the result. The MAT consisted of 200 trials for a total of 6480 s.

The total duration of the experiment was approximately 121 min, and the time for each trial was fixed for 2-BT (2 s) and MAT (32.4 s), respectively. All stimuli were displayed on a computer screen in white font and a black background. No feedback was provided to participants, regardless of the accuracy of the trial results. Participants were informed to emphasize the accuracy and speed of the results of the trials.

### 2.3. EEG Data Acquisition and Preprocessing

EEG signals were recorded from 64 Ag/AgCl electrodes based on the international 10–20 system. During the whole experiment, the electrode impedance remained below 5 KΩ throughout the recordings. The reference electrodes were the right and left mastoids. Both horizontal and vertical electrooculograms (HEOG and VEOG) were recorded from electrodes placed at the outer canthi as well as above and below the right eye. A widely used EEG preprocessing step was adopted, which included re-reference the average of all electrodes, band-pass filtering of 0.3–30 Hz, downsampled to 200 Hz, and independent component analysis (ICA) for artifact removal. The Adjust plugin algorithm was used to determine the artifact components and combined with the time-domain waveform and brain topography of each independent component for manual removal.

The duration of the MAT was 6480 s, which was much longer than the 2-BT. Moreover, we held that the effects of task switching on the brain would not last long. Therefore, 400 s EEG data were selected after the beginning and before the ending of the MAT, respectively (denoted as Pre_MAT, Post_MAT hereafter). It should be noted that in the following data analysis, the task-transformed EEG data used were Pre_2BT to Pre_MAT, and Post_MAT to Post_2BT. In addition, the collected EEG data was 64 electrodes, but 60 electrodes were used in the data analysis, CB1, CB2, HEO, and VEO were discarded. Subsequently, the EEG data involved in task conversion were decomposed into four standard bands by zero-phase digital filtering: delta (0.5–4 Hz), theta (4–8 Hz), alpha (8–13 Hz) and beta (13–30 Hz).

### 2.4. Network Features Estimation

The brain is composed of various regions, and different regions have their own functions. Cognitive functions are mainly derived from the interconnection between different brain regions [14,27,28]. Functional connectivity is built on the notion of perceiving brain regions as networks and quantifying the interactions of brain regions by measuring statistical coupling between nodes [28,29,30]. A sliding window approach was employed in the current work for the construction of functional connections. Here, the window length was 4 s with an overlap of 2 s, and 199 windows could be extracted from 400 s EEG data. Within each window for every frequency band, the functional connections between any two channels were estimated using the Phase lag index (PLI). As a phase-based functional connection method, PLI can be used to measure the synchronization degree of two-channel signals. The value range of PLI is 0 to 1, and a higher value indicates a stronger degree of phase synchronization between the two signals [31]. The number of functional connections per window was 60 × (60 − 1)/2 × 4 = 7080. Study has shown that network features can reflect the brain’s local separation and global integration of information processing capabilities [32].

The average of the functional connections of all windows corresponding to each sample, task process, and cadence were calculated. Functional connectivity contained a lot of complex information, but most of them were meaningless. The sparsity method was used to keep the connections whose connection strength was greater than the threshold as valid connections. It could ensure that the evaluation of network features would not be biased by different numbers of connections and possible low-weight false connections [33,34]. In this study, weighted networks, in which the values of the edges in the weighted network are the PLI values, were constructed with a sparsity of 15–30% and an increment of 1% for each step. We calculated the graph-theoretic properties of the brain network under different sparsity, and the spatial topology of functional connection was evaluated. Network features investigated the graph-theoretic properties of functional connection from two aspects: local differentiation and global integration respectively.

Weighted characteristic path length ($L^{w}$), weighted clustering coefficient ($C^{w}$), weighted global efficiency ($E_{global}^{w}$), and weighted local efficiency ($E_{local}^{w}$) are the network characteristics applied in this study. In complex networks, the path length is defined as the number of connected edges on the path from node i to node j. It usually has one or more paths, but researchers are interested in the shortest path, which is the best path for transmitting information [35]. In a weighted network, the weighted shortest path length between nodes can be calculated by taking the reciprocal of the weight, and the formula is shown in the Equation (1), where $I_{ij}^{W}$ denotes the length of the shortest path between node i and j, N is the set of all nodes in the network, and n is the number of nodes in the network.

(1) $$L^{w}=\frac{1}{n\left(n-1\right)}\underset{i\neq j\in N}{\sum }l_{ij}^{w}$$

The clustering coefficient reflects the possibility that all neighbor nodes of a node in the network are neighbors to each other, and it is an important parameter to measure the degree of internal grouping and connection tightness in complex networks. The weighted clustering coefficient is defined as Equation (2), where $t_{i}^{w}$ is the number of connections which is the weighted geometric mean of triangles around adjacent node i, and $k_{i}$ is the number of connected nodes of i.

(2) $$C^{W}=\frac{1}{n}\underset{i\in N}{\sum }\frac{2t_{i}^{w}}{k_{i}\left(k_{i}-1\right)}$$

As an improved network metric, global efficiency is closely related to the average characteristic path length and is a comprehensive indicator to measure the speed of information transfer in complex networks [36]. The shorter the average characteristic path length in a complex network, the greater its global efficiency and the higher the overall transmission rate between network nodes. Weighted global is defined as Equation (3), where $I_{ij}^{w}$ represents weighted shortest path length between node i and node j.

(3) $$E_{global}^{W}=\frac{1}{n\left(n-1\right)}\underset{i\neq j\in N}{\sum }\frac{1}{l_{ij}^{w}}$$

Local efficiency can measure the transmission ability of local information in complex networks and reflect the ability of complex network structures to resist attacks to a certain extent [24]. The definition of weighted local efficiency is shown in Equation (4), where $K_{i}$ is the degree of node i in binary networks. $l_{jh}^{w}\left(N_{i}\right)$ is the shortest path length between node j and node h passing through node i in weighted networks.

(4) $$E_{local}^{w}=\frac{\sum_{i\neq j\neq h\in N}(w_{ij}w_{jh}{\left[l_{jh}^{w}\left(N_{i}{)}^{-1}\right]\right)}^{1/3}}{2k_{i}\left(k_{i}-1\right)}$$

As mentioned previously, the sparsity in this study ranged from 15% to 30%. The area under the curve (AUC) approach was employed to avoid network parameter changes caused by different sparsity, so as to better characterize the changing trend of network characteristics [37,38]. Moreover, one-way analysis of variance (ANOVA) was carried out to determine the significant statistical differences in network features between Pre_2BT and Pre_MAT, Post_MAT and Post_2BT, Pre_2BT and Post_2BT.

### 2.5. Key Functional Connections Selection

PLI function connections with significant differences between Pre_2BT and Pre_MAT were selected (*p* < 0.05). The same procedure was applied between Post_MAT and Post_2BT. Then, the support vector machine recursive feature elimination (SVM-RFE) was used to rank the functional connections with statistical differences based on their PLI values. Specifically, the functional connections with statistical differences were ranked between Pre_2BT and Pre_MAT, and between Post_MAT and Post_2BT, respectively. SVM-RFE is a sequential backward selection algorithm based on the maximum interval principle of SVM, which is frequently used to calculate and rank the weight of each feature [39]. Radial Basis Function (RBF) was used as kernel function in SVM. The basic idea of SVM is to map the input features to a high-dimensional space after a nonlinear variation, and then find the optimal classification hyperplane in the high-dimensional space that maximizes the distance between two classes of samples [40]. The discriminant function of SVM is $f\left(x\right)=W^{T}x+b$, where $W={\left[w_{1},\;w_{2},\ldots ,w_{p}\right]}^{T}$ is the weight vector and b is the scalar. The mechanism of SVM is to satisfy the minimization of $P\left(W,\xi \right)$ in Equation (5), where *C* is the penalty factor used to achieve a compromise between the complexity of the algorithm and the proportion of misclassified samples. It should be noted that Equation (5) subjects to (6), and the value of $y_{i}$ is 1 or −1.

(5) $$P\left(W,\xi \right)=\frac{1}{2}{\left\|W\right\|}^{2}+C\overset{n}{\underset{i=1}{\sum }}\xi_{i}$$

(6) $$y_{i}\left[W^{T}x_{i}+b\right]\geq 1-\xi_{i},\;\xi_{i}\geq 0,\;i=1,\ldots ,n$$

SVM-RFE was originally proposed for the binary classification problem. The squared $w_{j}^{2}\;\left(j=1,\ldots ,p\right)$ of the weight vector W obtained from Equation (5) is used as the weight for feature ranking. The feature with the largest weight has the greatest amount of information. A feature with the lowest weight is removed in each iteration of SVM-RFE, and the SVM classifier is retrained again until the feature ranking is completed. In fact, the magnitude of $w_{j}^{2}$ corresponds exactly to the approximate variation of the criterion in Equation (5), that is $J=\frac{1}{2}{\left\|W\right\|}^{2}+C\overset{n}{\underset{i=1}{\sum }}\xi_{i}$. The optimal brain damage algorithm is used to calculate the change in *J* when the *j*-th feature is removed [41]. The criterion *J* can be expanded to the second order in the Taylor series as in Equation (7). On the optimal solution of *J*, only the second order is considered, so that $△J\left(j\right)\approx {\left(△w_{j}\right)}^{2}$. It can be seen from Equation (8) that removing the feature with the smallest $w_{j}^{2}$ will result in the smallest increase in *J* while improving the generalization performance. The aim of SVM-RFE is to find the subset of genes that produce the minimum standard.

(7) $$△J\left(j\right)=\frac{\partial J}{\partial w_{j}}△w_{j}+\frac{\partial^{2}J}{\partial^{2}w_{j}}{\left(△w_{j}\right)}^{2}+O\left({\left(△w_{j}\right)}^{3}\right)$$

(8) $$J\left(j\right)\approx J+w_{j}^{2}$$

**Figure 2:**
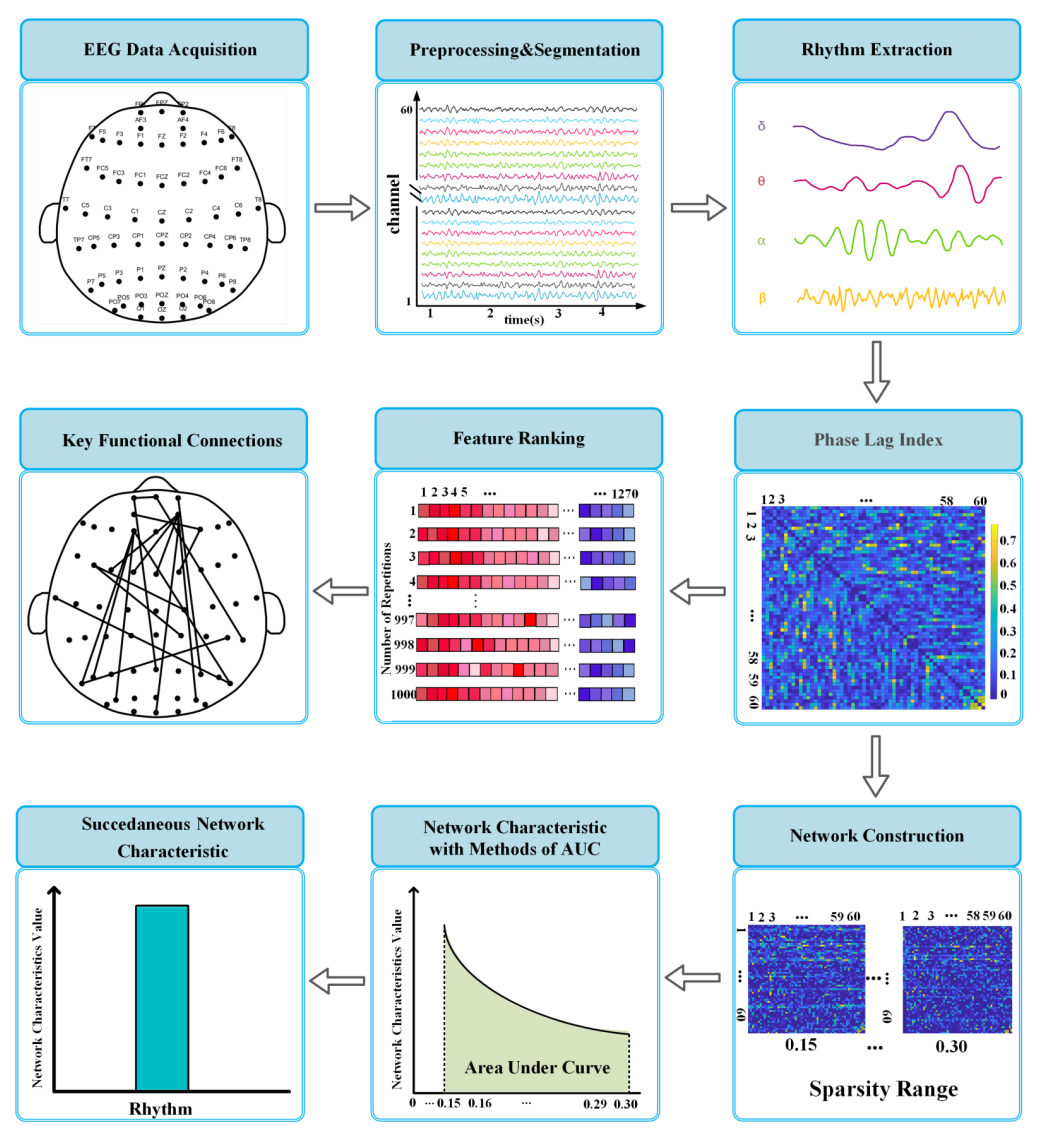
A flowchart of the feature extraction procedures.

In this study, for the ranking of functional connections, the weights of the features were calculated by the sorting criteria of the SVM-RFE internal classification model. The feature with the lowest weight would be removed. The same steps were executed for the rest features to remove the feature that had the lowest impact on the classification until all features were removed. To improve the robustness of ranking, we performed 1000 times of feature sorting with 90% samples used every time, resulting in 1000 × *n* ranked feature set (*n* is number of the statistically significant features in each rhythm). The final ranked feature set is obtained through counting (that is, the most common feature in the first column is the first significant feature; the most common feature in the first and second column is the second significant feature, and so on). All the above algorithms were implemented using MATLAB 2019b (Mathworks Inc., Natick, MA, USA). The schematic flow chart of the data analysis approach is shown in Figure 2.

## 3. Results

### 3.1. Mental Fatigue Determination

**Figure 3:**
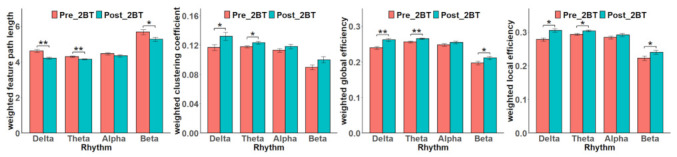
Network topology characteristics of four different rhythms during Pre_2BT and Post_2BT tasks, error bars are the standard deviation divided by the root number 20. * Indicates *p* < 0.05, ** indicates *p* < 0.01.

The 2-BT behavioral data accuracy and response time (RT) were collected from the 20 participants. No statistical difference was revealed between the Pre_2BT and Post_2BT. As expected, with the implementation of the MAT, the participants’ accuracy degraded, and reaction time increased under mental fatigue. Network characteristics of four different rhythms during Pre_2BT and Post_2BT tasks are shown in Figure 3. $L^{W}$, $E_{global}^{W}$, and $E_{local}^{W}$ were statistically different in delta, theta and beta. $C^{W}$ had statistical difference between delta and theta rhythms. In the classification of Pre_2BT and Post_2BT using four rhythms of PLI features, three machine learning algorithm models, SVM, random forest (RF) and K-Nearest Neighbor (KNN) were applied with a 10-fold cross-validation method, respectively. The highest accuracy was 98% with SVM in the Pre_2BT and Post_2BT classification was obtained. In recent studies, Wang et al. extracted features from EEG signals and fused them with EMG feature values to do a study on mental fatigue recognition for cognitive tasks and obtained an average accuracy of 95.32% [42]. Liu et al. conducted a classification of different levels of mental fatigue using several statistical features and machine learning algorithms with a 93% accuracy [43]. Compared to other studies of mental fatigue induced by cognitive tasks, there was a higher accuracy rate, which reflected that the participants had been put into a state of mental fatigue [42,43,44]. It was shown that the EEG signals can be more objective and accurate responses to the subject’s mental fatigue. These above findings indicated that mental arithmetic successfully induced mental fatigue.

### 3.2. Graph-Theoretical Network Analysis

**Figure 4:**
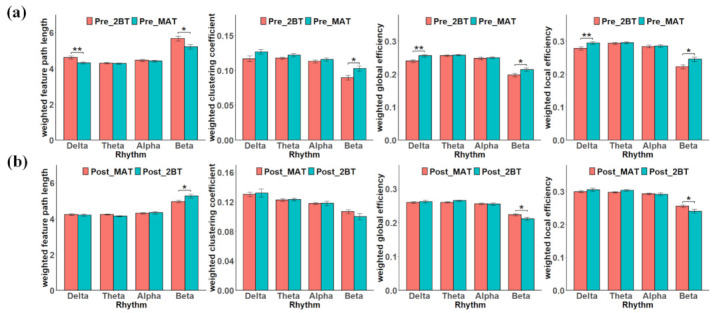
Four network topological features of different rhythms in task switching before and after mental fatigue. (**a**) for Pre_2BT to Pre_MAT; (**b**) for Post_MAT to Post_2BT processes. The error bars are the standard deviation divided by the root number 20. * Indicates *p* < 0.05, ** indicates *p* < 0.01.

Four brain network features of different rhythms in task switching before and after mental fatigue are shown in Figure 4. Figure 4a is the comparison of network features of Pre_2BT and Pre_MAT before mental fatigue task switching, and Figure 4b is Post_MAT and Post_2BT after mental fatigue. In Figure 4a, $L^{W}$, $E_{global}^{W}$, and $E_{local}^{W}$ had statistical differences in delta and beta rhythms and $C^{W}$ was only statistically different in beta rhythm. The statistical difference in delta rhythm was more obvious than that in beta. Furthermore, each rhythm of $C^{W}$, $E_{global}^{W}$, $E_{local}^{W}$ increased and that of $L^{W}$ decreased. The trends of theta and alpha were not obvious compared with delta and beta rhythms, and the change of network characteristics was consistent in four rhythms.

When Post_MAT was converted to Post_2BT, among the $L^{W}$, $E_{global}^{W}$, $E_{local}^{W}$ network features, only the beta rhythm had a statistical difference. In $C^{W}$, $E_{global}^{W}$ and $E_{local}^{W}$, the network eigenvalues of delta and theta increased during task switching, and the beta rhythm decreased. In $L^{W}$, the values of delta and theta rhythms decreased, but that of alpha and beta rhythms increased. During switching tasks after mental fatigue, the trend of beta rhythm in the four network features was opposite to that of delta and theta rhythms.

### 3.3. Selected Key Connectivity Features

**Figure 5:**
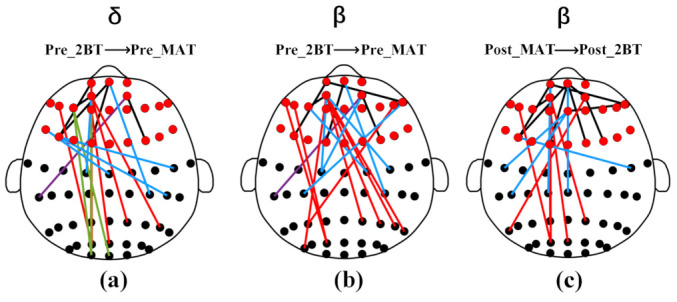
(**a**,**b**) are twenty functional connections for delta and beta rhythms during Pre_2BT conversion to Pre_MAT, respectively; (**c**) is the twenty beta functional connections extracted from Post_MAT transformation to Post_2BT. Frontal lobe leads are marked with red dots. The black edges indicate from the frontal region to the frontal region, the red edges refer to the frontal region to the parietal region, the green edges represent from the frontal region to the occipital region, the blue edges indicate from the frontal region to the central region, and the purple edges refer to the frontal region to the temporal region.

In the study of network features, delta and beta rhythms were statistically different during task switching before mental fatigue, and only beta rhythm was statistically different in task switching after mental fatigue. Considering the findings of network features, we conducted an in-depth study of statistically significant delta and beta rhythms in task switching before mental fatigue and beta rhythm after mental fatigue. The top 20 functional connections calculated by SVM-RFE feature sorting were selected respectively and the topography of selected functional connections is shown in Figure 5.

Figure 5a is the selected functional connections of delta rhythm during the conversion of Pre_2BT to Pre_MAT. All functional connections are related to the frontal region. Specifically, they are mainly distributed in the frontal to the frontal region (five connections), frontal to the central region (five connections) and frontal to the parietal region (six connections). In addition, fewer functional connections are distributed in the frontal to the occipital region (three connections), and the frontal to the temporal region (one connection). The selected function connection values of Pre_2BT are all smaller than those of Pre_MAT.

Functional connections of beta rhythm selected during the transition from Pre_2BT to Pre_MAT are shown in Figure 5b. The connections related to the frontal region in the beta rhythm account for 100% of the total connections. More specifically, the distribution of functional connections was from frontal to frontal region (five connections), frontal to central region (six connections), frontal to parietal region (eight connections) and frontal to temporal regions (one connection). For the twenty functional connections selected during the transition from Pre_2BT to Pre_MAT before mental fatigue, the PLI values of Pre_2BT were all smaller than those of Pre_MAT.

The functional connections of beta rhythm during the transition from the Post_MAT task to Post_2BT are shown in Figure 5c. There are nine functional connections from the frontal region to the frontal region, five functional connections from the frontal region to the central region, and six functional connections from the frontal region to the parietal region. The same as the conversion before mental fatigue, all the functional connections were the connections between the frontal region and other brain regions. In addition, the PLI values of the twenty functional connections in the Post_MAT process were all greater than Post_2BT.

## 4. Discussion

In this study, two mental tasks (2-BT and MAT) were used to investigate the changes in brain function during task switching before and after mental fatigue. The findings are as follows: First, the 2-BT before and after MAT was statistically different in network features, and the classification accuracy based on PLI features was as high as 98%. Second, delta and beta rhythms exhibited significant differences in network features during task transitions before mental fatigue. In addition, each rhythm of $C^{W}$, $E_{global}^{W}$, $E_{local}^{W}$ increased and that of $L^{W}$ decreased, along with the enhancement of functional connections between the frontal region and other regions. Third, task switching from MAT to 2-BT after mental fatigue, the trend of change in network characteristics of beta rhythm was opposite to that before mental fatigue. Furthermore, only in beta rhythm there were statistical differences.

The behavioral data recorded in Pre_2BT and Post_2BT tasks showed that participants performed Post_2BT with increased reaction time and decreased accuracy compared to Pre_2BT. It indicated the effectiveness of MAT in inducing mental fatigue [45]. In addition, delta, theta and beta rhythms showed significant differences in 2-BTs before and after MAT and the $L^{W}$ of each rhythm decreased significantly. A study related to driving mental fatigue found that $L^{W}$ significantly decreased in the later stage of the driving experiment compared with the warm-up period, which is in accordance with our finding [46]. The decrease in the shortest path represents an increase in brain efficiency, a direct reason for which task proficiency is related to task switching [34]. An incremental positive correlation with mental fatigue level was found in $C^{W}$, $E_{global}^{W}$ and $\;E_{local}^{W}$. It can be deduced that the brain was more efficient at performing Post_2BT, with enhanced interactions in neural populations.

Network features can reflect the functional integration and local aggregation of information between brain regions when performing mental tasks [32,45,47]. In this study, we analyzed brain network reorganization during task switching before and after mental fatigue. Delta and beta rhythms were found to be significantly different in task switching before mental fatigue and the difference in delta was more obvious. Zarjam et al. found that the delta rhythm related to the level of working memory load [48]. In a time-frequency analysis of EEG data from participants performing card sorting tasks, the results showed that delta power responded strongly to task-switching and novel distractor pairs [49]. Combined with the findings of these studies, it can be found that delta rhythm is correlated with cognitive tasks and mental fatigue and have the potential to serve as mental workload classification. In this study, $C^{W}$, $E_{global}^{W}$ and $\;E_{local}^{W}$ showed a consistent increase with the conversion of Pre_2BT to Pre_MAT, and a significant decrease in $L^{W}$. Dai et al. found that the functional connectivity network density of theta rhythm increased, and the characteristic path lengths decreased significantly after the 0-back task was switched to the 2-BT [50]. Consistent with our findings, task switching prior to mental fatigue can correlate with an increase in the overall transmission rate between brain network nodes connection density, which can be a reallocation of brain resources and alleviate inertia.

In the study of Post_MAT conversion to Post_2BT in a state of mental fatigue, $L^{W}$, $E_{global}^{W}$ and $\;E_{local}^{W}$ showed statistical differences only in beta rhythm. Several studies have supported such findings that beta rhythm increases with cortical arousal and characteristic path length is also positively correlated with task duration [51,52]. The variation trends of the delta, theta, and alpha rhythms in network features were not obvious. However, the beta rhythm was more variable and opposite to the trend of task switching before mental fatigue. The findings indicate that delta, theta, and alpha rhythms are insensitive to task switching in mentally fatigued states, and beta rhythm can be well used for the detection of task variations. Relevant studies have proved that the fluctuation of beta rhythm is related to cognitive function [51,53]. Moreover, among the network features, alpha and beta were found to have opposite changing trends compared to delta and theta. The weak changes in network features exhibited by low-frequency rhythms are associated with attentional inhibition when switching tasks in a mentally fatigued state [54,55]. To sum up, the low-frequency EEG rhythm will have stronger task inertia and less sensitive response to task changes than the high-frequency EEG rhythms under mental fatigue.

Statistical analysis of selected top 20 delta and beta rhythm functional connections before and after mental fatigue found that all selected connections were related to frontal regions, which are known to be related to attention by several studies [56,57,58]. In addition, the functional connections of delta and beta in the frontal and central areas, frontal and parietal areas occupied a large proportion in the task switching. Studies have shown that these two regions were considered to be part of the so-called “frontal-parietal network” or “cognitive control network” responsible for executing a wide range of executive functions related to goal-directed behavior [59,60]. In essence, the changes in task complexity and cognitive demand load were reflected in the frontal connections. The study found that the strength of inferior frontal and superior temporal lobe connections was positively correlated with cognitive load [61], and our findings also corroborate this observation.

During the task switching before mental fatigue, the functional connections across brain regions accounted for 55%, but the cross-brain connections of beta rhythms only accounted for 20% after mental fatigue. We speculated that mental fatigue may inhibit dynamic interactions between different brain regions [44,62]. After switching from Pre_2BT to Pre_MAT, the strength of functional connections in the delta and beta bands increased, which could be considered as phase synchronization enhancement between the different channels. When Post_MAT was converted to Post_2BT, the strength of all selected PLI function connections decreased. Kakkos et al. observed increased PLI intensity at high loading levels in most feature-extracted connectivity features except occipital connectivity features, which corroborates our study [62]. In the process of switching from 2-BT to MAT, MAT required the communication and cooperation of various brain regions, so there was an increased demand for cognitive resources. The increase in connection strength could reflect this. Conversely, after MAT switched to 2-BT, the cognition demanded to perform 2-BT decreased, and the synchrony between channels decreased. The decrease in synchronization may be an indication of autonomous control over the task performed, or a reallocation of cognitive resources [21,63,64].

Several studies have shown that in the process of preprocessing, the selection of reference electrodes and the removal of ICA-based artifacts will affect the quality of the data and lead to inaccurate experimental results [65,66]. The specificity between different participants will also affect the results. In addition, what we need to consider is that although the two tasks used in this study required continuous cognitive updating at a higher workload, but the participants felt MAT to be slightly more difficult than the 2-BT during the experiment. It should be noted that the original intention of our experiment did not include irrelevant stimuli or distraction effects. During the experiment, individual differences in effort, attention, and relevant cultural knowledge may have some influence on the results, which should be avoided in our future work. Besides, in future research, we hope to set the task difficulty during task switching as a variable. Specifically, both tasks with balanced difficulty and tasks with diverse difficulty will be explored. The findings of future research will be compared with those of the present study to gain more insight into the neural mechanisms of the brain during task switching.

## 5. Conclusions

In this study, we extracted the functional connections and network features during the conversion process of two cognitive tasks (2-BT and MAT) and used the SVM-RFE feature ranking algorithm to select the top twenty functional connections. The results showed that mental fatigue could be successfully induced by MAT. We found that the network characteristics of theta and beta rhythms changed significantly in the task transition before mental fatigue and the PLI values increased, which indicated the efficiency of the brain’s task execution increased. In the task switching after mental fatigue, the beta rhythm was statistically different, and the changing trend was opposite to that before mental fatigue. In contrast, the low-frequency rhythm network characteristics showed little variation. The network characteristics of beta rhythm can be used as a neural marker of task switching before and after mental fatigue. Overall, this study is instructive for studying the neural mechanism of task switching before and after mental fatigue.

## Data Availability

Not applicable.
